# “Rotation Overlap Method” for 3D Wiring in Chronic Total Occlusion

**DOI:** 10.1016/j.jaccas.2024.102937

**Published:** 2025-01-08

**Authors:** Calvin Leung, Cheuk Bong Ho, Ivan Wong, Esmond Fong, Michael Kang Yin Lee, Alan Ka Chun Chan

**Affiliations:** Division of Cardiology, Department of Medicine, Queen Elizabeth Hospital, Hong Kong SAR

**Keywords:** 3D wiring, chronic total occlusion, rotation overlap method

## Abstract

We describe the “rotation overlap method” for 3-dimensional (3D) wiring without orthogonal projections. This method ultilizes “parallax” to resolve wire shaft (WS) and target (TG) relationships according to the ‘rotation overlap rule’. Rotate the fluoroscopic view toward the WS: if the WS-TG are seen closer, the WS becomes anterior to the TG (vice versa [away from WS = posterior]). This rule applies to any fluoroscopic rotation under 180°, regardless of axial or oblique to vessel. The “WS-TG closer” criterion replaces orthogonal projections to constrain the WS-TG spatial relationship. The steps for 3D wiring are as follows: For 2 fluoroscopic projections, the view with WS-TG closer is the “closer view,” and the other is the “far view”. 1) Resolve WS-TG anterior/posterior relationship in the “closer view” based on whether fluoroscopy is rotated toward/away from the WS in the “far view” to reach the “closer view.” 2) Optimize the “closer view” for WS-TG overlap. 3) In the optimized “closer view,” rotating the wire in the correct direction until the wire tip (WT) is central will precisely direct the WT toward the TG. Four scenarios (WS anterior/posterior × WT right/left) determine the correct wire rotation (clockwise or counter-clockwise).

## Graphical Abstract

**Figure:**
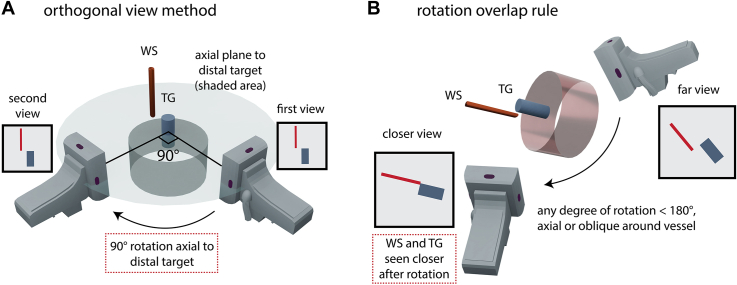

Traditional 3-dimensional (3D) wiring for chronic total occlusion (CTO) uses 2 orthogonal fluoroscopic projections to reconstruct wire shaft (WS) and wire tip (WT) positions relative to target.^1^, ^2^, ^3^ For antegrade wiring to distal target, this method improves wire control, minimizes unnecessary movement, and reduces subintimal hematoma, enhancing success and safety.^3^ However, it is complex due to difficulties in obtaining precise orthogonal projections in different vessel anatomy and mentally reconstructing 3D images of both WS and WT from 2-dimensional views.^2^

**Take-Home Messages**

• The “rotation overlap method” enables 3D wiring without orthogonal projections.
• This method adapts to vessel anatomy, allows flexible fluoroscopic use while maintaining 3D information, precisely directs the WT, and simplifies operator decision making.

To address these challenges, we introduce the “rotation overlap method,” a modified approach to resolve WS-target (TG) relationships and guide correct wire movement in 2 nonorthogonal planes.

## The “Rotation Overlap Rule”

We propose using parallax-induced changes in observed WS-TG distance after fluoroscopic rotation, combined with rotation direction relative to WS-TG, to determine their spatial relationship. This "rotation overlap rule" states the following:

Rotate fluoroscopic view toward WS: if the WS-TG are seen closer, the WS becomes anterior to TG; and vice versa [away from WS = posterior].

This rule applies to any fluoroscopic rotation <180°, regardless of axial or oblique rotation relative to the vessel (Video 1). To clarify, it determines the WS-TG relationship using 2 static fluoroscopic projections in which WS-TG appears closer in one view, not rotational angiography.

**Figure 1:**
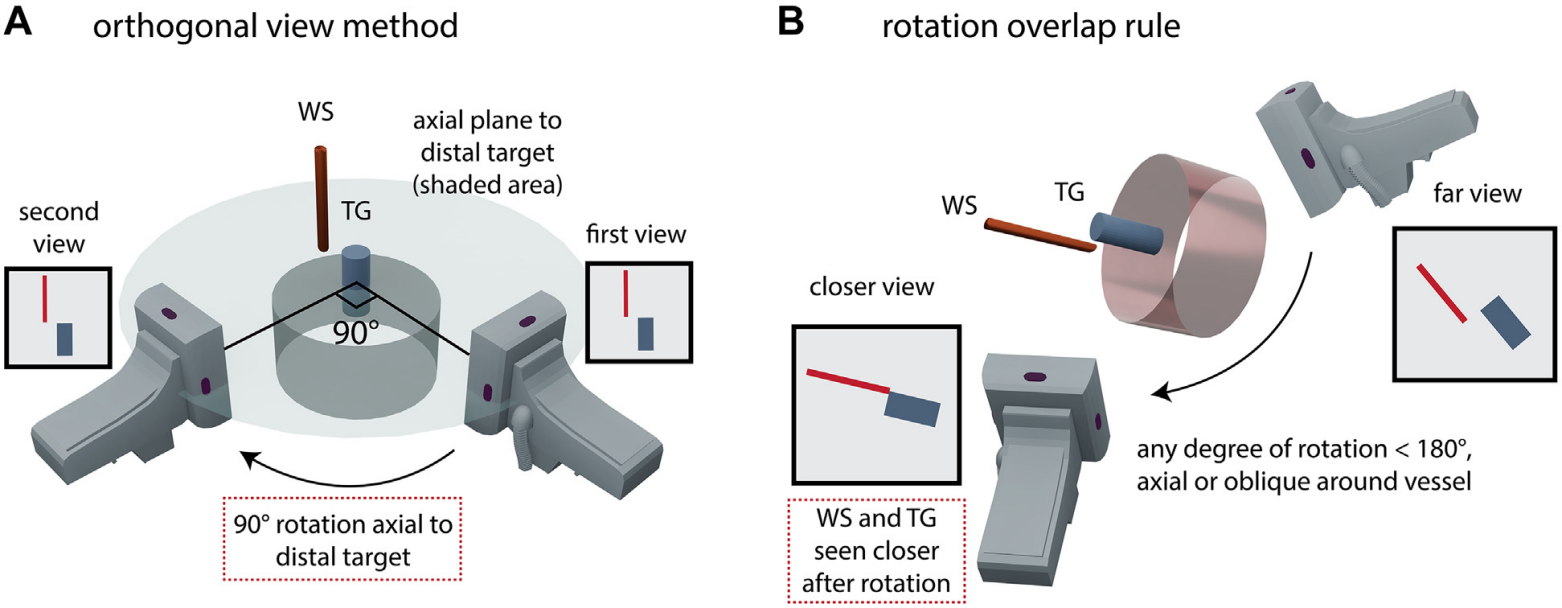
Conceptual Differences Between the Orthogonal View Method and the Rotation Overlap Rule (A) The orthogonal relationship between the 2 views constrains the wire shaft (WS) position (relative to the target [TG]) in the second view, based on its position in the first view and the direction of fluoroscopic rotation. (B) The parallax-induced “WS-TG seen closer” criterion constrains the WS position (relative to TG) in the second (closer) view, based on its position in the first (far) view and the direction of fluoroscopic rotation, replacing the need for orthogonal projections.

The criterion of “WS-TG seen closer” after fluoroscopic rotation serves as the key constraint to WS-TG relationship, replacing the need for orthogonal projections (Figure 1, Video 2). The distinction between the orthogonal view method and the “rotation overlap rule” to resolve WS-TG relationship is summarized in Table 1.

**Table 1:** Comparison of the Orthogonal View Method and “Rotation Overlap Rule” for Resolving Spatial Relationship Between WS and TG

	Orthogonal View Method	Rotation Overlap Rule
Principle	Based on orthogonal projections	Based on the phenomenon of parallax
Key constraint to WS-TG relationship in the second view	The second view is orthogonal to the first	The WS-TG are seen closer in the second (closer) view compared with the first (far) view
Method of key constraint fulfilment	Use predetermined optimal fluoroscopic angles according to the vessel segment	Direct visualization of “WS-TG closer” by fluoroscopy
Rotation plane	Axial to distal target	Can be axial or oblique to distal target
Rotation degree	90° rotation	Any degree <180°
Number of fluoroscopic views required	Two orthogonal views	Two views with one of them showing “WS-TG closer.” A third view will be required if initial WS-TG distance appear similar in 2 views.
Applicable view	Can resolve WS and TG relationship in both orthogonal views	Can resolve WS and TG relationship only in the “closer view”
Relationship of fluoroscopic rotation direction to WS position after rotation	Rotate fluoroscopic view 90° axial to distal target toward WS, WS becomes anterior to target in the next view Vice versa (away from WS = posterior)	Rotate fluoroscopic view (any degree, axial, or oblique to distal target) toward WS, if WS-TG is seen closer, WS becomes anterior to target in the closer view Vice versa (away from WS = posterior)
Freedom in fluoroscopic rotation	Restricted to 90° apart views along the axial plane of distal target	Relatively unrestricted as long as a clear “closer view” can be obtained
Stability of WS-TG A/P relationship	WS-TG A/P relationship fragile when they are observed far apart and require precise orthogonal projection	WS-TG A/P relationship stable as only the closer view is used
Source of error	Fluoroscopic rotation not axial to distal target	Misinterpretation of relative WS-TG distance

A/P = anterior/posterior; TG = target; WS = wire shaft.

**Figure 2:**
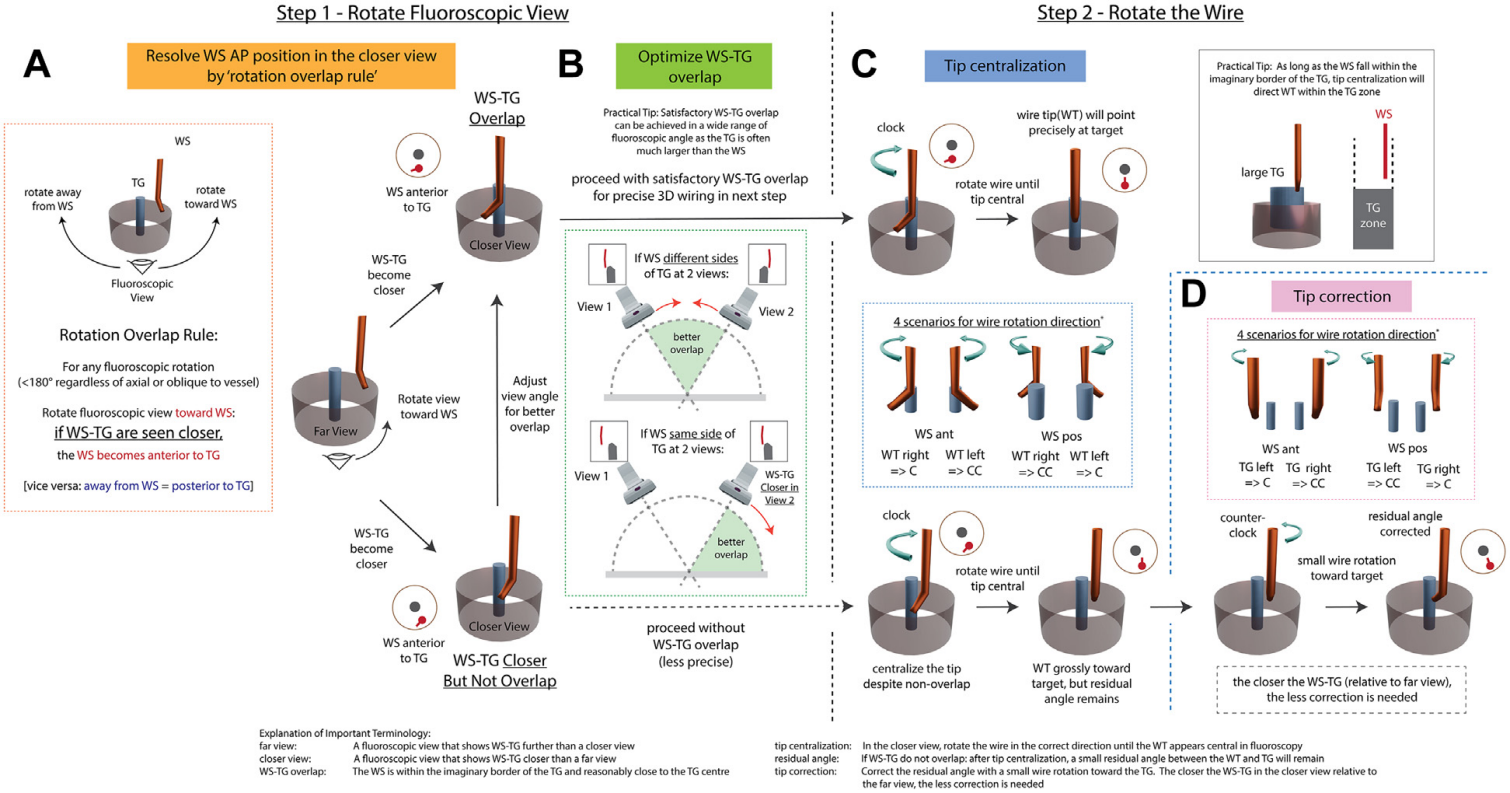
Rotation Overlap Method (A) Use the "rotation overlap rule" to determine the WS A/P position in the closer view. Rotate the fluoroscopic view toward the WS: if the WS-TG are seen closer, the WS becomes anterior to TG, and vice versa (away from WS = posterior). This rule works for any fluoroscopic rotation <180° around the vessel, regardless of axial or oblique. The WS-TG A/P relationship in the closer view is determined by whether the fluoroscopy is rotated toward or away from the WS in the far view to reach the closer view. (B) Optimize WS-TG overlap: If the WS is at different sides of the TG in 2 views, choose a view in-between for better overlap. If on the same side, move farther in the direction of the closer view. (C) Tip centralization: There are 4 scenarios of WS (A/P) in relation to WT (right/left) to determine correct wire rotation (clockwise or counter-clockwise). When WS-TG overlap, continue wire rotation until the WT is central in fluoroscopy to point precisely to the TG. (D) Tip correction: Without WS-TG overlap, tip centralization will result in the WT grossly pointing toward the TG, with a small residual angle. Correct this with a slight wire rotation toward the TG. The rotation direction (clockwise or counter-clockwise) is intuitive based on WS position (A/P) and TG location (right/left). ∗Wire rotation direction shown for antegrade wiring. A/P = anterior/posterior; WT = wire tip; other abbreviations as in Figure 1.

The “rotation overlap method,” a step-by-step application of this rule to determine correct wire movement, is described in the following section and illustrated in Figure 2.

## Procedural Steps

### Determine the WS-TG anterior/posterior relationship in the closer view

In a pair of fluoroscopic views, the one with WS-TG seen closer is the “closer view,” and the other is the “far view.” The WS-TG anterior/posterior (A/P) relationship in the closer view is determined by whether the fluoroscopy is rotated toward or away from the WS in the far view to reach the closer view (Figure 2A, Video 1). The rotation overlap method uses only the WS A/P position information of the closer view to guide wire manipulation toward the TG.

### Optimization of WS-TG overlap

Before wire movement, optimize the closer view by maximizing the WS-TG overlap (Figure 2B, Video 3): Start with 2 views on opposite sides of the midline. If the WS is at different sides of the TG, choose a view in-between for better overlap. If the WS is on the same side in both views, adjust fluoroscopy farther toward the side with the closer WS-TG appearance. A satisfactory overlap is typically possible across a wide range of angles, as TG is usually much larger than WS.

### Perform wire rotation

Once the WS-TG overlapping is optimized, perform wire rotation in the optimized closer view. Knowing the WS A/P position, use the wire tip (WT) direction (right/left) to decide the correct rotation (clockwise or counter-clockwise) to reach TG. There are 4 scenarios (Figure 2C, Video 4). With WS-TG overlap, continue rotating the wire until the WT is centralized will precisely direct it toward the TG (tip centralization) (Figure 2C). This method has a clear fluoroscopic endpoint, making it simple and precise, unaffected by non-1:1 wire torque transfer.

When WS-TG overlapping is not possible, the operator can still proceed with wire movement, accepting reduced precision. First, perform tip centralization as described previously, resulting in the WT grossly pointing toward TG, with a small residual angle (Figure 2C). Correct this with a slight wire rotation toward the TG (tip correction) (Figure 2D). The rotation direction (clockwise or counter-clockwise) is intuitive based on WS position (A/P) and TG location (right/left) (Figure 2D, Video 5). The closer the WS-TG in the closer view compared with the far view, the smaller the correction needed.

We believe this “rotation overlap method” to 3D wiring offers several advantages:

1. Allows 3D wiring without orthogonal projections, which could be difficult to obtain depending on vessel segment.
2. Determines WS-TG relationship largely independent of vessel orientation or fluoroscopic rotation (degree or axis) as long as a clear “closer view” can be obtained. This may facilitate 3D wiring in varying vessel anatomy or relatively tortuous vessel segments.
3. Takes advantage of the stability of WS-TG A/P relationship in the closer view, unlike in the far view where their relative A/P position can change with a small degree of fluoroscopic rotation.
4. Allows relatively unrestricted fluoroscopic rotation to optimal working views while preserving 3D spatial information.
5. Enables precise WT direction toward the TG by optimizing WS-TG overlap in the closer view.
6. Uses a fluoroscopic endpoint for tip centralization, resolving issues with non-1:1 wire torque transfer and removing the need to work out the exact WT position or the degree of rotation.
7. Uses simple decision making: the operator determines WS A/P position in the closer view, then observes WT direction (right/left) to choose rotation direction (4 possibilities), reducing mental processing.
8. The operator can choose not to optimize the WS-TG overlap if high precision is not the priority. The closer view naturally directs the WT grossly toward the TG during tip centralization, with further refinement possible through tip correction.

## Case Examples

We demonstrate 3D wiring using the “rotation overlap method” during distal cap puncture in 2 CTO cases of right coronary artery and left circumflex artery (LCX).

### Case 1

A patient with refractory angina was referred for right coronary artery CTO intervention. Bilateral injection was performed for angiographic analysis (Supplemental Figure 1A) and distal target was visualized via ipsilateral and contralateral collateral filling. Antegrade wiring with XTA/Gladius EX (Asahi Intecc) failed to penetrate the hard CTO body. With vessel course relatively marked by calcium, we used the Gaia Next 3 (Asahi Intecc) to wire close to the distal cap. We advanced the antegrade microcatheter, rather limited by calcium in the CTO body. We used the rotation overlap method for distal cap puncture.

**Figure 3:**
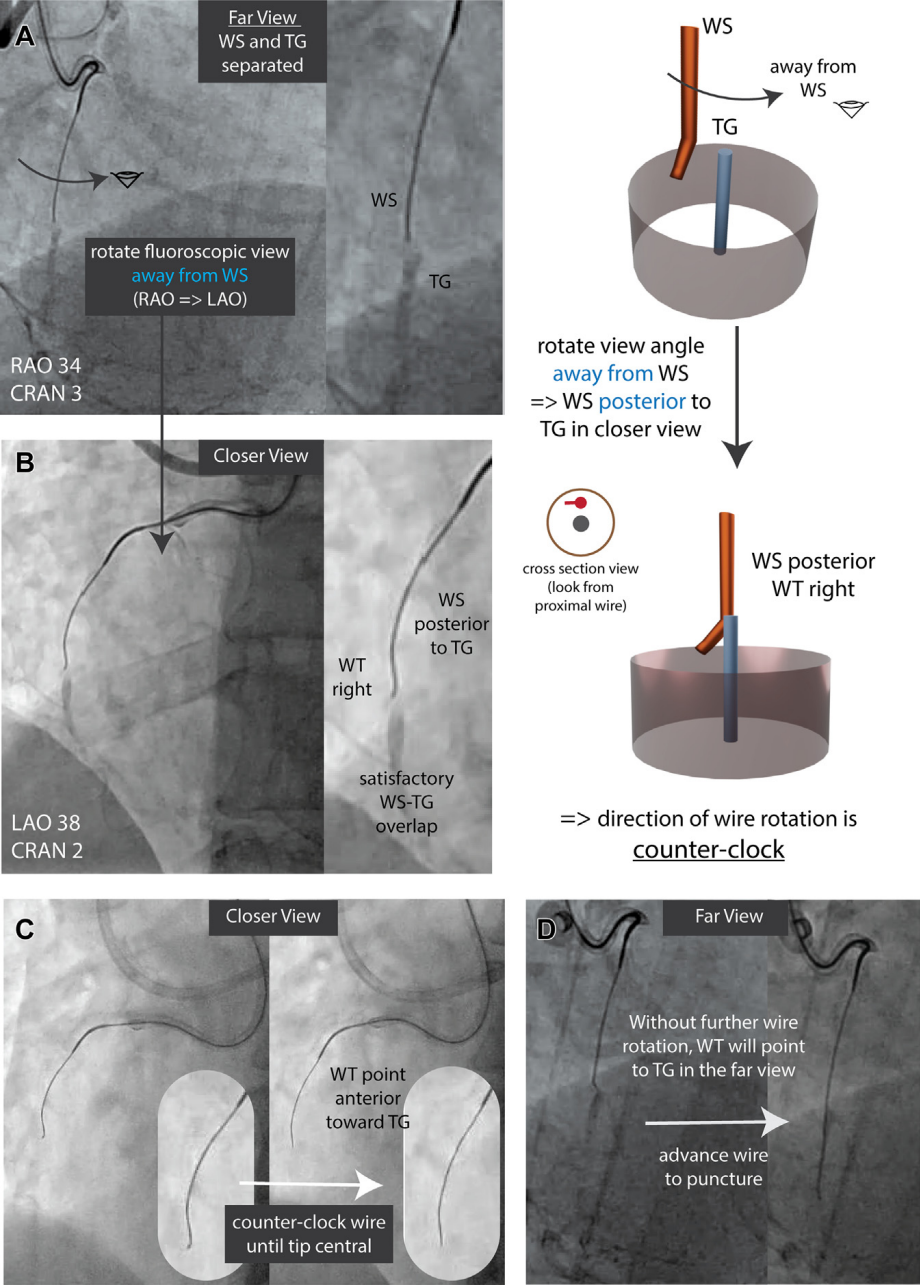
Example of Rotation Overlap Method for Distal Cap Puncture in Right Coronary Artery CTO (A, B) Apply the “rotation overlap rule” to determine WS A/P position in the closer view. Rotating away from the WS in the far view (RAO) indicates WS is posterior to the TG in the closer view (LAO). Achieve satisfactory WS-TG overlap. With WS posterior and WT right, the correct wire rotation is counter-clockwise to reach the TG. (C) Tip centralization: In the closer view (LAO), rotate the wire counter-clockwise until the WT appears central, directing it anteriorly toward the TG. (D) Verify WT direction in the far view without additional rotation. Advance the wire to puncture the distal cap. CAUD = caudal; CRAN = cranial; CTO = chronic total occlusion; LAO = left anterior oblique; RAO = right anterior oblique; other abbreviations as in Figures 1 and 2.

We performed 2 nonorthogonal fluoroscopic projections in right anterior oblique (RAO) and left anterior oblique (LAO) views (Figures 3A and 3B). The WS-TG appeared closer in the LAO (closer view) than RAO (far view). Rotating away from WS in the far view toward the closer view indicated WS was posterior to TG in the closer view (Figure 3B). With satisfactory WS-TG overlap in the LAO view, no further optimization was needed (Figure 3B).

As the WT pointed right and WS was posterior, we rotated the wire counter-clockwise until the tip centralized in the closer view, pointing anteriorly toward the TG (Figure 3C). We confirmed correct WT direction pointing to the TG in the far view without further wire rotation (Figure 3D). The wire was then advanced and entered the distal true lumen on the first attempt (Figure 3D). The final angiographic result after stenting was satisfactory (Supplemental Figure 1B).

### Case 2

A patient was referred for LCX CTO intervention due to persistent angina. Bilateral injection was performed for angiographic analysis (Supplemental Figure 2A) and distal target was visualized via contralateral collateral filling. Antegrade wiring with XTA entered the proximal cap, but both XTA and Gladius EX failed to wire through the hard, calcific CTO body. With vessel course marked by calcium, we stepped up to the Conquest Pro 9 (Asahi Intecc) and wired through the CTO body. We then switched to 3D wiring with the rotation overlap method for distal cap puncture.

**Figure 4:**
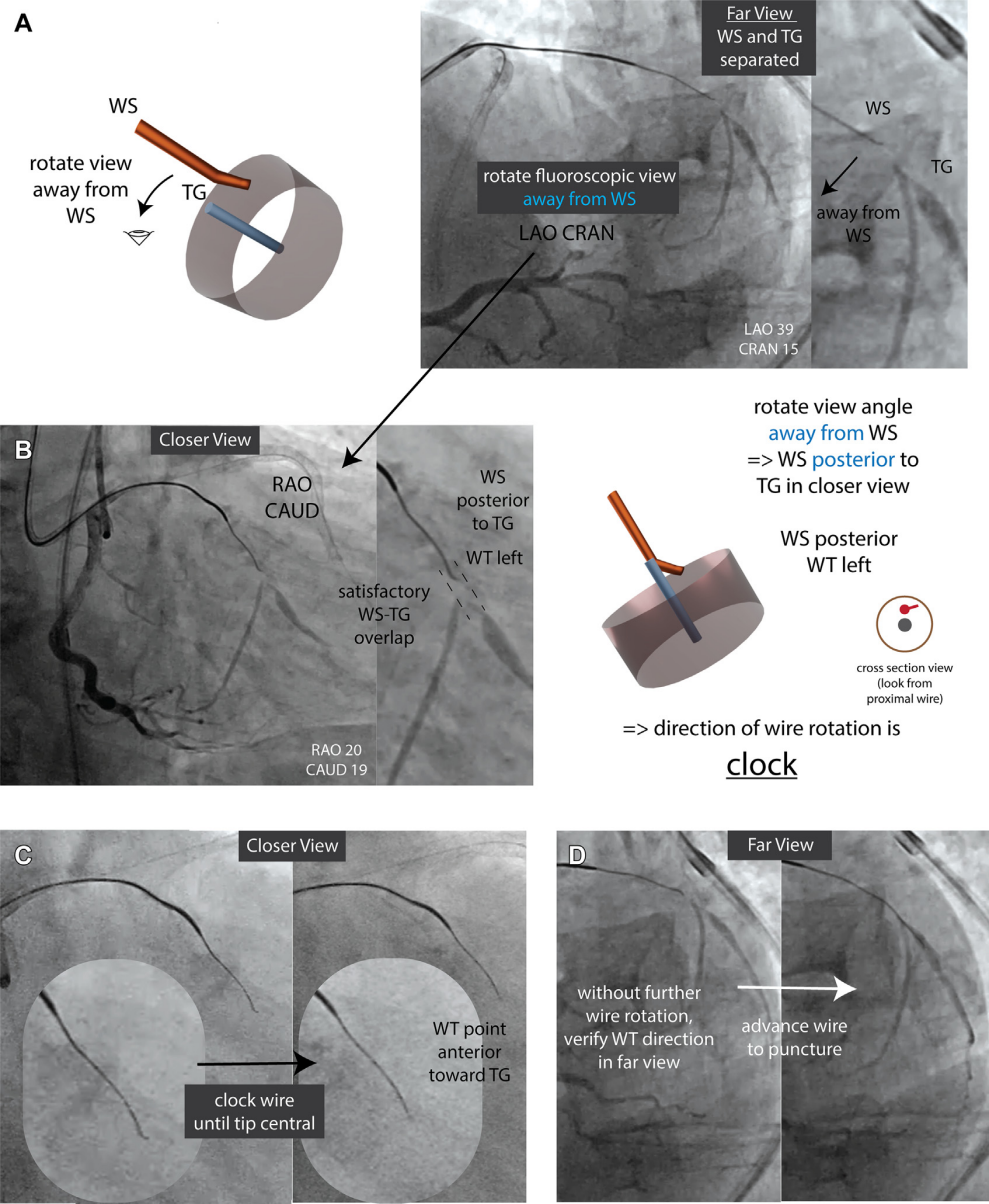
Example of Rotation Overlap Method for Distal Cap Puncture in Left Circumflex Artery CTO (A, B) Apply the “rotation overlap rule” to determine WS A/P position in the closer view. Rotating away from the WS in the far view (LAO CRAN) indicates the WS is posterior to the TG in the closer view (RAO CAUD). Achieve satisfactory WS-TG overlap. With WS posterior and WT left, the correct wire rotation is clockwise to reach the TG. (C) Tip centralization: In the closer view (RAO CAUD), rotate the wire clockwise until the WT appears central, directing it anteriorly toward the TG. (D) Verify WT direction in the far view without additional rotation. Advance the wire to puncture the distal cap. Abbreviations as in Figure 1, Figure 2, Figure 3.

We obtained 2 nonorthogonal fluoroscopic projections: RAO caudal (closer view) and LAO cranial (far view) (Figures 4A and 4B). Rotating away from the WS in the far view to the closer view indicated WS is posterior to TG in the closer view (Figure 4B). WS-TG overlap was satisfactory without further optimization (Figure 4B).

With WS posterior and WT pointing left in the closer view, we rotated the wire clockwise until the tip centralized, pointing anteriorly toward the TG (Figure 4C). We confirmed WT direction in the far view without further wire rotation, then advanced the wire to puncture the distal cap (Figure 4D). The wire entered the distal true lumen on the first attempt. The final angiographic result was satisfactory (Supplemental Figure 2B).

Video 6 provides a step-by-step video recording of the rotation overlap method during distal cap puncture in this LCX CTO case.

## Potential Pitfalls

Apply the rotation overlap method with the fundamental principles of 3D wiring, as described by Wu et al.^1^ These include initiating 3D wiring at an appropriate distance from the distal TG (Supplemental Figure 3A) and using high-penetration, directable guidewires with adequate backup support. Even with precise WT direction, "near-side miss" and "far-side overshoot miss" can occur due to varying longitudinal and lateral distances between the wire and distal TG (Supplemental Figures 3B and 3C). For near-side miss, pull the wire back farther to allow longer travel distance toward the target. For far-side overshoot, retract the wire to above the distal TG, then rotate the wire ∼180° to redirect the WT toward the TG. In both situations, the rotation overlap method can be reapplied to ensure WT direction before reattempting puncture.

General limitations of 3D wiring include poor distal target visualization, inadequate wire torque control, impenetrable tissue, and severe tortuosity. These factors can limit the technique applicability and should be considered when selecting candidates.

## Conclusions

We described the “rotation overlap method” to enable 3D wiring without orthogonal projections. We demonstrated its step-by-step application during distal CTO cap puncture with clinical cases. This method adapts to vessel anatomy, allows flexible fluoroscopic use while maintaining 3D information, precisely directs the WT, and simplifies operator decision making.

## Funding Support and Author Disclosures

The authors have reported that they have no relationships relevant to the contents of this paper to disclose.

## Appendix

Video 1 Rotation Overlap Rule

Video 2 How ‘WS-TG Seen Closer’ Constrains Their Spatial Relationship

Video 3 Optimizing WS-TG Overlap

Video 4 With WS-TG Overlap: Tip Centralization

Video 5 Without WS-TG Overlap: Tip Correction After Tip Centralization

Video 6 Case Recording With Step-by-Step Illustration
