# Supplementary material for: “Rotation Overlap Method” for 3D Wiring in Chronic Total Occlusion
Source: JACC Case Rep. 2025 Jan 8;30(6):102937. doi: 10.1016/j.jaccas.2024.102937 (PMC12011170; doi:10.1016/j.jaccas.2024.102937)
Supplement: Supplemental Figures 1-3 [file mmc7.docx]

Supplemental Figure 1 – Case of RCA CTO


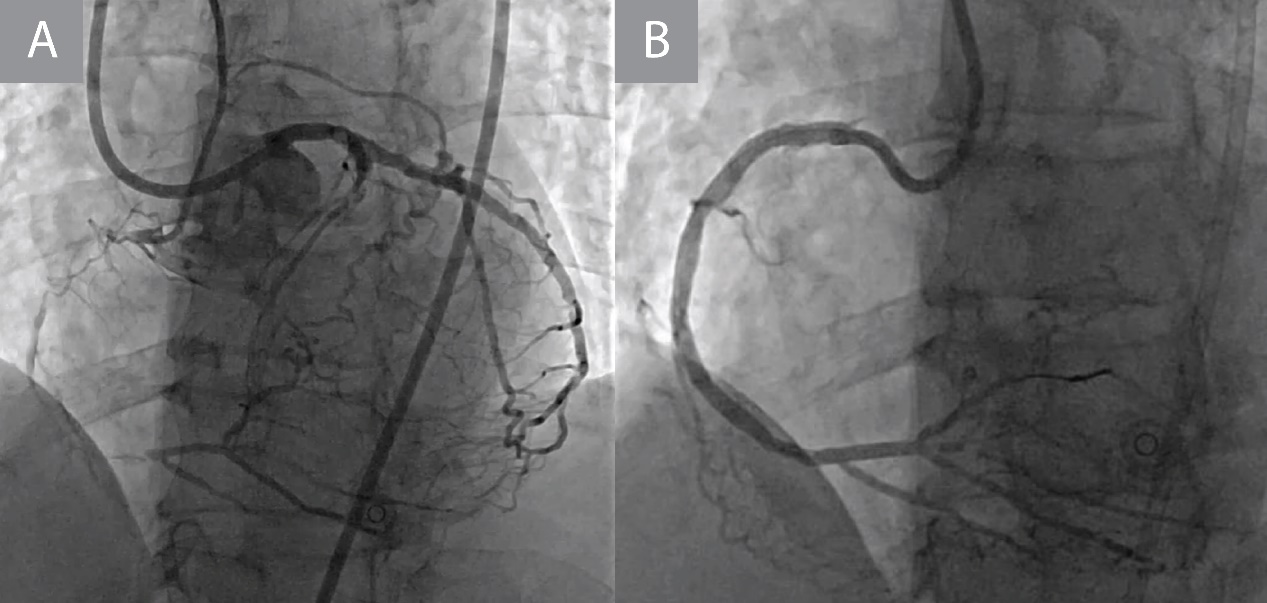


(A) baseline angiogram with bilateral injection

(B) final angiographic result

Supplemental Figure 2 – Case of LCX CTO


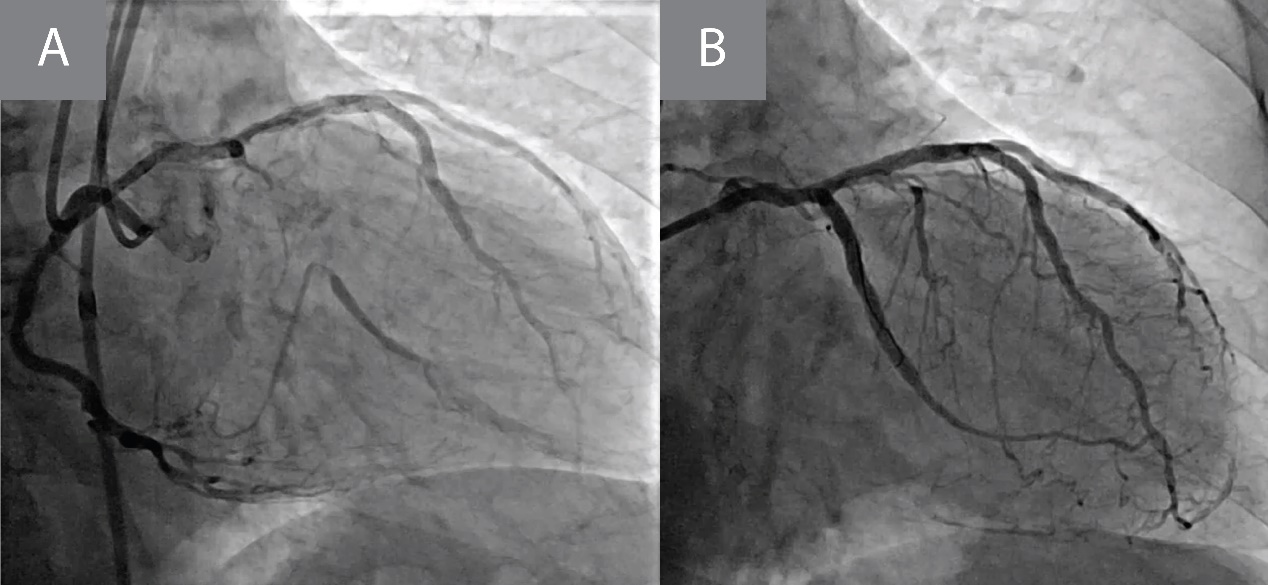


(A) baseline angiogram with bilateral injection

(B) final angiographic result

Supplemental Figure 3 – Importance of appropriate distance between distal wire and target in 3D wiring


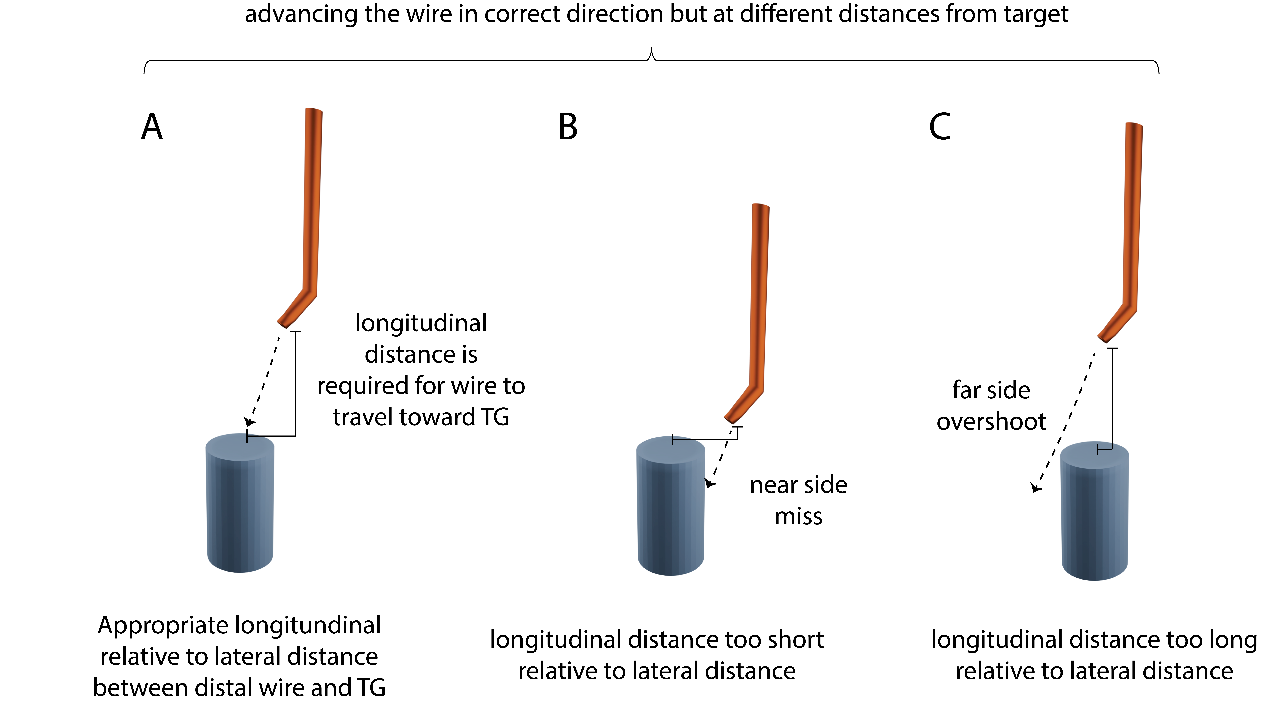


(A) Appropriate longitudinal distance is required for the wire to travel in the correct direction toward target.

(B) Longitudinal distance too short relative to lateral distance results in 'near-side miss'.

(C) Longitudinal distance too long relative to lateral distance results in 'far-side overshoot'.

Abbreviations: TG (target)
